# Supplementary material for: Long-Term Serological Investigations of Influenza A Virus in Free-Living Wild Boars (Sus scrofa) from Northern Italy (2007–2014)
Source: Microorganisms. 2022 Sep 1;10(9):1768. doi: 10.3390/microorganisms10091768 (PMC9506564; doi:10.3390/microorganisms10091768)
Supplement: Supplementary file 1 [file microorganisms-10-01768-s001.zip › micorganisms-1855141-Supplementary.pdf]

Table S1. Hemagglutination inhibition assay (HI) titers obtained from 141/148 sera of wild boars (*Sus scrofa*) screened as positive for influenza A virus nucleoprotein (NP) by NP-ELISA (Northern Italy, 2007-2014). Three recaptured wild boars (ID 2623, ID 2940, ID 2955) were included in this table.

The HI assays were performed using as antigens the following strains of inactivated influenza A viruses:
H1N1/98, A/swine/Italy/1513/1998/H1N1av; H1N1/13, A/swine/Italy/311368/2013/H1N1av;
H1N1pdm/13, A/sw/Italy/282866/2013/H1N1pdm;
H1N2/98, A/swine/Italy/1521/1998/H1huN2; H1N2/2009, A/swine/Italy/284922/2009/H1huN2; H3N2/98, A/swine/Italy/1523/1998/H3N2; H3N2/13, A/swine/Italy/311349/2013/H3N2.

Sampling date	WB serum ID	Age class*	Sex	HI titers from wild boars tested IAV positive by NP-ELISA						
				H1N1/98	H1N1/13	H1N1pdm/13	H1N2/98	H1N2/09	H3N2/98	H3N2/13
9 March 2007	1984	U	U	—	—	—	—	—	—	1:20
5 April 2007	2035	2	F	—	—	—	—	—	—	1:20
17 April 2007	2056	2	F	—	—	—	—	—	—	—
	2058	2	M	—	—	—	—	—	—	—
22 May 2007	2124	U	M	—	—	—	—	—	—	—
	2125	3	M	—	—	—	—	—	—	1:20
	2130	1_1st sc	M	—	—	—	—	—	—	—
	2132	4	M	—	—	—	—	—	—	—
23 May 2007	2134	1	F	—	—	—	—	—	—	—
25 May 2007	2140	2	F	—	—	—	—	—	—	—
29 May 2007	2144	3	F	—	—	—	—	—	—	—
	2146	1_1st sc	M	—	—	—	—	—	—	—
	2147	1_1st sc	M	—	—	—	—	—	—	—
	2149	3	F	—	—	—	—	—	—	—
	2150	1_1st sc	F	—	—	—	—	—	—	—
	2152	1_1st sc	F	—	—	—	—	—	—	—
	2154	1_1st sc	F	—	—	—	—	—	—	—
1 June 2007	2156	4	M	—	—	—	—	—	—	—
	2158	4	F	—	—	—	—	—	—	—
5 June 2007*	2164	U	U	—	—	—	—	—	—	—
	2165	U	U	—	—	—	—	—	—	—
	2166	U	U	—	—	—	—	—	—	—
	2169	U	U	—	—	—	—	—	—	—
7 June 2007	2172	2	F	—	—	—	—	—	—	—
12 June 2007	2175	U	U	—	—	—	—	—	—	1:40
	2179	1_1st sc	M	—	—	—	—	—	—	—
	2180	1_1st sc	M	—	—	—	—	—	—	1:20
	2181	1_1st sc	M	—	—	—	—	—	—	—
	2182	1_1st sc	F	—	—	—	—	—	—	—
	2183	1_1st sc	F	—	—	—	—	—	—	—
14 June 2007	2186	1_1st sc	M	—	—	—	—	—	—	—
	2187	1_1st sc	F	—	—	—	—	—	—	1:20
	2188	1_1st sc	M	—	—	—	—	—	—	—
	2189	1_1st sc	M	—	—	—	—	—	—	—
	2190	1_1st sc	F	—	—	—	—	—	—	—
	2191	1_1st sc	F	—	—	—	—	—	—	—
	2192	1_1st sc	M	—	—	—	—	—	—	—
	2194	1_1st sc	M	—	—	—	—	—	—	—
	2195	1_1st sc	M	—	—	—	—	—	—	—
	2197	U	U	—	—	—	—	—	—	1:20
	2198	U	U	—	—	—	—	—	—	—
	2199	1_1st sc	F	—	—	—	—	—	—	—
	2201	1_1st sc	F	—	—	—	—	—	—	—
	2203	1_1st sc	M	—	—	—	—	—	—	—

Table S1. *Cont.*

Sampling date	WB serum ID	Age class*	Sex	HI titers from wild boars tested IAV positive by NP-ELISA						
				H1N1/98	H1N1/13	H1N1pdm/13	H1N2/98	H1N2/09	H3N2/98	H3N2/13
15 June 2007	2204	3	F	—	—	—	—	—	—	—
26 June 2007	2222	1_1st sc	M	—	—	—	—	—	—	1:20
3 July 2007	2230	1_1st sc	M	—	—	—	—	—	—	1:20
	2231	1_1st sc	F	—	—	—	—	—	—	1:20
29 August 2007	2311	1_1st sc	M	—	—	—	—	—	—	—
13 September 2007	2333	1_2nd sc	M	—	—	—	—	—	—	—
20 November 2007	2421	1_1st sc	M	—	—	—	—	—	—	—
19 February 2009	2590	2	M	nd	nd	nd	nd	nd	nd	nd
	2591	4	M	nd	nd	nd	nd	nd	nd	nd
24 February 2009	2596	U	M	nd	nd	nd	nd	nd	nd	nd
	2598	3	M	nd	nd	nd	nd	nd	nd	nd
26 February 2009	2600	2	M	1:320	1:160	1:80	—	—	—	—
	2601	2	F	1:160	1:80	1:40	—	—	—	—
	2602	1_2nd sc	M	1:40	1:80	1:40	—	—	—	—
	2603	1_2nd sc	F	1:80	1:40	1:40	—	—	—	—
	2604	1_2nd sc	M	1:40	1:40	1:20	—	—	—	1:20
	2606	3	F	1:80	1:80	1:40	—	—	—	1:20
27 February 2009	2608	2	M	1:320	1:320	1:80	—	—	—	1:20
4 March 2009	2611	2	M	1:40	1:40	1:20	—	—	—	—
	2612	2	M	1:160	1:160	1:40	—	—	—	1:20
	2613	2	F	1:80	1:40	1:20	—	—	—	1:20
	2614	2	M	1:20	1:20	—	—	—	—	1:40
10 March 2009	2623 (§)	2	M	1:80	1:40	1:40	—	—	—	1:20
	2624	2	F	1:80	1:40	1:40	—	—	1:20	1:40
12 March 2009	2627	2	M	1:160	1:80	1:40	—	—	—	1:40
	2628	4	F	1:640	1:640	1:160	—	—	—	—
	2629	2	M	1:40	1:20	1:20	—	—	1:20	1:40
17 March 2009	2632	3	M	1:40	1:40	1:20	—	—	—	—
18 March 2009	2634	2	M	1:40	1:20	1:40	—	—	—	1:20
20 March 2009	2635	3	F	1:40	1:40	1:20	—	—	—	1:40
24 March 2009	2637	3	F	1:80	1:40	1:40	—	—	—	1:40
	2638	U	F	1:40	1:40	1:40	—	—	—	—
	2640	2	M	1:80	1:40	1:20	—	—	—	1:20
2 April 2009	2646	3	F	1:40	1:40	1:20	—	—	—	—
7 April 2009	2651	U	U	1:20	1:20	1:20	—	—	—	1:80
	2652	U	U	1:40	1:40	1:20	—	—	—	1:20
8 April 2009	2654	2	M	1:40	1:40	—	—	—	—	1:20
	2656	2	M	1:40	1:40	1:40	—	—	—	1:20
21 April 2009	2660	3	F	1:80	1:80	1:40	—	—	—	1:20
24 April 2009	2669	U	U	1:20	1:40	1:20	—	—	—	1:40
29 April 2009	2671	2	M	1:40	1:40	1:80	—	—	—	—
	2672	2	F	1:80	1:40	1:40	—	—	—	—
	2673	2	F	1:80	1:80	1:40	—	—	—	—
	2674	3	F	1:160	1:80	1:80	—	—	—	—
	2675	3	M	1:80	1:80	1:40	—	—	—	—
7 May 2009	2690	4	F	1:40	1:20	—	—	—	—	—
12 May 2009	2707	3	M	1:20	1:20	1:20	—	—	—	1:40
19 May 2009	2711	2	M	1:80	1:40	1:40	—	—	—	—
30 June 2009	2754	2	M	1:320	1:80	1:40	—	—	—	—
2 July 2009	2756	3	M	1:80	1:40	1:40	—	—	—	—
	2757	2	F	1:160	1:80	1:80	—	—	—	—

Table S1. *Cont.*

Sampling date	WB serum ID	Age class*	Sex	HI titers from wild boars tested IAV positive by NP-ELISA						
				H1N1/98	H1N1/13	H1N1pdm/13	H1N2/98	H1N2/09	H3N2/98	H3N2/13
16 July 2009	2781	3	F	1:80	1:80	1:40	—	—	—	—
30 September 2009	2809	4	M	1:160	1:80	1:160	—	—	—	—
20 October 2009	2829	3	F	1:320	1:160	1:80	—	—	—	1:20
	2832	3	F	1:160	1:80	1:40	—	—	—	—
	2833	3	F	1:160	1:80	1:40	—	—	—	—
24 November 2009	2868	2	F	—	—	—	—	—	—	—
26 November 2009	2870	2	F	—	—	—	—	—	—	—
11 December 2009	2894	3	M	1:320	1:80	1:40	—	—	—	—
7 May 2010	2940 (§)	3	M	1:640	1:640	1:320	—	—	—	—
18 May 2010	2955 (§)	4	M	1:160	1:80	1:40	—	—	—	—
	2957	U	U	1:160	1:80	1:40	—	—	—	1:20
19 May 2010	2959	3	F	1:160	1:160	1:160	—	—	—	—
31 May 2010	3002	3	F	1:160	1:160	1:80	—	—	—	—
4 June 2010	3007	3	F	1:640	1:320	1:320	—	—	—	—
	3019	1_1st sc	F	1:40	1:20	1:20	—	—	—	—
18 June 2010	3044	3	M	1:1280	1:320	1:320	—	—	—	—
27 July 2010	3092	4	M	1:40	1:40	1:20	—	—	—	—
3 September 2010	3104	3	F	1:80	1:40	1:40	—	—	—	1:40
16 February 2011	3165	3	F	nd	nd	nd	nd	nd	nd	nd
31 May 2011	3297	1_2nd sc	F	—	—	—	—	—	—	—
6 June 2011	3303	3	M	—	—	—	—	—	—	1:20
14 June 2011	3322	3	F	1:20	—	—	—	—	—	—
15 July 2011	3361	1_2nd sc	F	—	—	—	—	—	—	1:20
18 July 2011	3363	3	M	—	—	—	—	—	—	—
23 July 2011	3376	3	F	1:320	1:160	1:40	—	—	—	—
7 October 2011	3507	4	F	1:80	1:40	1:40	—	—	—	—
11 November 2011	3576	4	M	1:80	1:20	1:20	—	—	—	—
1 June 2012	3803	4	F	1:160	1:80	1:40	—	—	—	—
21 June 2012	3806	3	M	—	—	—	—	1:40	1:20	1:80
12 September 2012	3838	1_2nd sc	M	nd	nd	nd	nd	nd	nd	nd
19 September 2012	3849	1_2nd sc	M	—	—	—	—	—	—	—
20 September 2012	3852	3	F	—	—	—	—	1:20	—	—
	3853	1_2nd sc	M	—	—	—	—	—	—	—
26 September 2012	3874	3	M	—	—	—	—	—	—	1:20
1 October 2012	3887	1_2nd sc	F	—	—	—	—	—	—	—
	3888	1_2nd sc	F	—	—	—	—	—	—	—
4 October 2012	3896	1_2nd sc	M	—	—	—	—	—	—	—
	3897	1_2nd sc	M	—	—	—	—	—	—	—
	3898	1_2nd sc	F	—	—	—	—	—	—	—
	3907	1_2nd sc	M	—	—	—	—	—	—	—
5 October 2012	3903	1_2nd sc	M	—	—	—	—	1:20	—	—
	3904	1_2nd sc	M	—	—	—	—	1:20	—	—
9 October 2012	3910	2	M	—	—	—	—	—	—	—
10 October 2012	3914	1_2nd sc	F	—	—	—	—	—	—	—
	3918	1_2nd sc	F	—	—	—	—	—	—	—
1 November 2012	3962	3	F	nd	nd	nd	nd	nd	nd	nd
20 November 2012	3999	3	M	—	—	—	—	—	—	—
4 March 2013	4091	4	F	1:20	1:20	1:20	—	—	—	—
4 June 2013	4269	2	M	—	—	—	—	—	—	—

Table S1. *Cont.*

Sampling date	WB serum ID	Age class*	Sex	HI titers from wild boars tested IAV positive by NP-ELISA						
				H1N1/98	H1N1/13	H1N1pdm/13	H1N2/98	H1N2/09	H3N2/98	H3N2/13
20 May 2014	4668	4	M	—	—	—	—	—	—	—
12 June 2014	4714	4	M	—	—	—	—	—	—	—
	4716	4	M	—	—	—	—	—	—	1:20
13 June 2014	4717	4	F	—	—	—	—	—	—	—

WB, wild boar; ID, identification; F, female; M, male; U, undetermined; 1_1st sc , 1 st subclass 1; 2nd sc , 2nd subclass 1; *, Age classes (in months): 0 mo.< age class 1 ≤6 mo.; 6 mo.< age class 2 ≤14 mo.; 14 mo.< age class 3 ≤24 mo.; age class 4 > 24 mo.; U, undetermined; ^, for statistical analysis age class 1 was further categorized into: 0 mo.<1 st subclass 1≤3 mo., and 3 mo.<2nd subclass 1≤6 mo; —, negative (<20 HI reciprocal titer); (§), recaptured wild boars showing seroconversions. Colored bars show animals caught in the same cage-trap, and color tones show wild boars captured by different cage-traps in the same day.
